# Concentration of vascular endothelial growth factor in the tumour tissue as a prognostic factor of soft tissue sarcomas

**DOI:** 10.1054/bjoc.2001.1837

**Published:** 2001-06

**Authors:** K Yudoh, M Kanamori, K Ohmori, T Yasuda, M Aoki, T Kimura

**Affiliations:** Department of Orthopaedic Surgery, Toyama Medical and Pharmaceutical University, Toyama, Japan

**Keywords:** microvessel density, vascular endothelial growth factor, overall survival, tumour recurrence, metastasis, soft tissuearcoma

## Abstract

Previous studies have shown that the prognosis of patients who have tumours with high microvessel density (MVD) is worse than that of patients who have a lower density in a variety of cancers. In this study, we investigated the clinical relevance of neovascularity assessed by MVD and the concentration of vascular endothelial growth factor (VEGF) in the tumour tissue of patients with soft tissue sarcoma in comparison with major clinicohistologic parameters by univariate and multivariate analysis. In 115 patients with soft tissue sarcoma, MVD was measured by counting vessels stained with factor VIII antibody. The concentration of VEGF in the tumour tissue was determined by enzyme-linked immunosorbent assay. These parameters were then compared with disease outcome. The concentration of VEGF in the tumour tissue, but not MVD, was found to be correlated with disease outcome in patients with soft tissue, sarcoma. VEGF concentration in the tumour tissue showed a relationship with the clinical stage and histologic grade of the tumour. There was no significant difference in the levels of tissue VEGF concentration and MVD among soft tissue sarcomas classified according to histologic type. The level of tissue VEGF concentration in patients who had subsequent local recurrence and metastasis were significantly higher than the respective values in patients who did not have such disease outcome. No significant correlation existed between MVD and the concentration of VEGF in the tumour tissue. Univariate analysis showed that a high tissue VEGF concentration was associated with poor overall survival of the patient and a greater probability that local recurrence and metastasis had occurred. Multivariate analysis revealed that the tissue concentration of VEGF is an independent prognostic factor for the disease outcome of patients with soft tissue sarcoma. VEGF concentration in the tumour tissue, but not MVD, is an additional prognostic parameter for disease outcome in patients with soft tissue sarcoma, regardless of histologic type. © 2001 Cancer Research Campaign http://www.bjcancer.com


*British Journal of Cancer* (2001) **84**(12), 1610–1615
© 2001 Cancer Research Campaign
doi: 10.1054/ bjoc.2001.1837, available online at http://www.idealibrary.com on IDEAL®

http://www.bjcancer.com

### Concentration of vascular endothelial growth factor in the tumour tissue as a prognostic factor of soft tissue sarcomas

**K Yudoh, M Kanamori, K Ohmori, T Yasuda, M Aoki and T Kimura**

Department of Orthopaedic Surgery, Toyama Medical and Pharmaceutical University, Toyama, Japan

**Summary** Previous studies have shown that the prognosis of patients who have tumours with high microvessel density (MVD) is worse than that of patients who have a lower density in a variety of cancers. In this study, we investigated the clinical relevance of neovascularity assessed by MVD and the concentration of vascular endothelial growth factor (VEGF) in the tumour tissue of patients with soft tissue sarcoma in comparison with major clinicohistologic parameters by univariate and multivariate analysis. In 115 patients with soft tissue sarcoma, MVD was measured by counting vessels stained with factor VIII antibody. The concentration of VEGF in the tumour tissue was determined by enzyme-linked immunosorbent assay. These parameters were then compared with disease outcome. The concentration of VEGF in the tumour tissue, but not MVD, was found to be correlated with disease outcome in patients with soft tissue, sarcoma. VEGF concentration in the tumour tissue showed a relationship with the clinical stage and histologic grade of the tumour. There was no significant difference in the levels of tissue VEGF concentration and MVD among soft tissue sarcomas classified according to histologic type. The level of tissue VEGF concentration in patients who had subsequent local recurrence and metastasis were significantly higher than the respective values in patients who did not have such disease outcome. No significant correlation existed between MVD and the concentration of VEGF in the tumour tissue. Univariate analysis showed that a high tissue VEGF concentration was associated with poor overall survival of the patient and a greater probability that local recurrence and metastasis had occurred. Multivariate analysis revealed that the tissue concentration of VEGF is an independent prognostic factor for the disease outcome of patients with soft tissue sarcoma. VEGF concentration in the tumour tissue, but not MVD, is an additional prognostic parameter for disease outcome in patients with soft tissue sarcoma, regardless of histologic type. © 2001 Cancer Research Campaign http://www.bjcancer.com

**Keywords**: microvessel density; vascular endothelial growth factor; overall survival; tumour recurrence; metastasis; soft tissue sarcoma

Several basic and clinical studies have indicated that the aggressiveness of solid tumours such as the growth, invasion, and metastatic potential is dependent on angiogenesis (Gimbrone et al, 1972; Liotta et al, 1974; Folkman 1990; Folkman et al, 1989). Vascularization supplies nutrition and oxygen to proliferating tumour cells (Gimbrone et al, 1974). Several reports have demonstrated a positive correlation between an increased number of new vessels and the development of metastasis (Gimbrone et al, 1974; Liotta et al, 1974; Liotta et al, 1991). It has been reported that neovasularity assessed by intratumoral microvessel density (MVD) correlates with clinicopathologic factors and the prognosis of a variety of cancers (Yamazaki et al, 1994; Maeda et al, 1995; Brawer, 1996; Gasparini et al, 1996; Takabayashi et al, 1996).

To date, many angiogenic factors have been identified as follows: basic fibroblast growth factor, vascular endothelial growth factor (VEGF), platelet-derived endothelial cell growth factor, hepatocyte growth factor, transforming growth factor, epidermal growth factor, tumour necrosis factor-α, interleukin-8 and so on. It is now thought that expression of the tumour cell derived angiogenic factors is specific to each tumour and dependent on the process of tumour growth and spreading. Several studies have noted that the level of expression of VEGF, which is a strong angiogenic factor, correlates with neovascularity and with tumour progression in human colon cancer (Takahashi et al, 1995), human brain tumour (Takano et al, 1996), human breast cancer (Maeda et al, 1996), and several experimental tumour models (Miller et al, 1994; Zhang et al, 1995; Claffey et al, 1996). VEGF secreted from tumours may contribute to tumour growth, invasion and metastasis not only via an autocrine pathway to tumour cells, but via a paracrine pathway to surrounding microvessels (Takahashi et al, 1995). Overall, determination of the degree of neoangiogenesis is emerging as one of the powerful prognostic tools.

Recent studies on a large number of soft tissue sarcoma cases have claimed that the grade of the tumour, size of the tumour and tumour depth are independent prognostic factors (Gaynor et al, 1992; Coindre et al, 1996). There is a general consensus that the size of tumour tissue is an important and easily obtainable parameter of tumour growth and that the growth of tumour is largely dependent on neovascularity in solid tumours. However, it remains unclear whether the level of angiogenic activity correlates with the prognosis of soft tissue sarcomas. Soft tissue sarcomas represent a heterogeneous group of relatively rare malignant tumours with many histologic types. Because angiogenesis is essential for tumour growth, the development of local recurrence and metastasis, tumour angiogenesis assessed by MVD and the level of VEGF expression may be good prognostic factors for soft tissue sarcomas, regardless of histologic type.

*Received 4 October 2000*
*Received 27 February 2001*
*Accepted 28 February 2001*

*Correspondence to:* K Yudoh

In the present study, to evaluate whether angiogenic activity has any impact on the prognosis of patients with soft tissue sarcoma, we investigated whether a relationship exists between MVD, the concentration of VEGF by enzyme-linked immunosorbent assay (ELISA) in the tumour tissue, and disease outcome.

### MATERIALS AND METHODS

### Patients and tumour specimens

The subjects consisted of patients who underwent follow-up care at Toyama Medical and Pharmaceutical University Hospital and cooperative Cancer Center Hospital for verified cases of soft tissue sarcoma between 1990 and 1996. All patients who met the following criteria were included in this study: (1) the patient had a newly diagnosed primary tumour; (2) the tumour was histologically classified as a soft tissue sarcoma; (3) the tumour was radically resectable; (4) the patient had no other disease that would influence angiogenesis, such as wound healing, rheumatoid arthritis, and diabetic retinopathy. 115 patients met the study criteria. These included 58 men and 57 women; the average age was 61.5 ± 20.4 y at diagnosis (mean ± standard deviation (SD); range, 28 to 77 y). The observation period ranged between 63 and 176 months (mean ± SD, 91 ± 11 months). Surgery with curative intent was performed in all of the patients. Simple local excision was performed in 12 patients (7.0%); wide resection in 95 patients (82.6%); and amputation in 8 patients (10.4%). Surgical treatment was preceded by radiotherapy in 13 patients; chemotherapy in 55 patients; and both radiotherapy and chemotherapy in 10 patients. After the surgery, 3 patients received radiotherapy, 7 patients received chemotherapy, and 5 patients received both radiotherapy and chemotherapy. All of the tumours were histologically confirmed. The histologic grade and clinical stage of soft tissue sarcoma were documented based on the criteria of the UICC TNM Classification of malignant tumours (Hermanek and Sobin 1992). Each tumour was graded as having a high, moderate and low malignancy grade using the National Cancer Institute grading system (Mandard et al, 1989). The characteristics of tumour patients are summarized in Table 1. All patients gave informed consent for participation in this study.

The resected tumour specimens were immediately fixed in 10% phosphate-buffered formalin for 48 hours and embedded in paraffin. Sections of 6 μm in thickness were made and mounted on glass slides. Other parts of tissues were immediately frozen with liquid nitrogen and stored at –80˚C. The tumour tissues were homogenated with a motor-driven Teflon pestle for 5 minutes on ice in 1 ml of extraction buffer (25 mM Tris (pH 7.4), 100 mM NaCl, 20 mM $NH_4HCO_3$) per 100 mg tissue wet weight, and the tissue extract obtained after centrifugation at 15 000 rpm for 20 minutes at 4˚C was aliquoted to a 200-μM vial, stored at –80˚C, and used for ELISA.

### Immunohistochemical staining

Tissue sections were deparaffinized and incubated with 10% normal goat serum in phosphate-buffered saline (PBS) for 30 minutes. The sections were incubated with 200 μg $ml^{-1}$ of anti-factor VIII mAb (1:50) (Dako, Santa Barbara, CA) for 60 minutes at room temperature. Purified mouse IgG (Cappel, West Chester, PA) at the same protein concentration as that in the tissue section was used as a negative control. The sections were incubated with biotin-conjugated goat anti-mouse (1:100, Santa Cruz Biotechnology, Santa Cruz, CA) for 10 minutes, followed by washing in PBS for 5 minutes, followed by washing in PBS for 5 minutes, and then stained with freshly prepared aminoethylcarbazole solution for 10 minutes, followed by a 5-minutes wash in tap water. The sections were counterstained with haematoxylin and mounted with aqueous mounting media.

### Microvessel counting

Each slide was studied for antigen expression by 2 investigators. Any single brown-stained cell which indicates an endothelial cell that stained for the presence of factor VIII or a cluster of cells clearly distinguishable from the background was counted as a vessel. Branching structures were counted as a single vessel, unless there was a break in the continuity of the structure. The stained sections were screened at 5 times magnification, to identify the areas of highest vascular density. Sclerotic areas where microvessels were sparse and areas immediately adjacent to benign tissue were not considered in the vessel count. After the area of highest neovascularization was identified, individual vessel counts were performed at × 200 magnification (0.739 $mm^2$ per field).

**Table 1** Characteristics of tumour patients

| Histological type | Number of patients | Counts | | |
|---|---|---|---|---|
| | | Grade of malignancy Low + Moderate / High | Size < 5 cm / ≥ 5 cm | Stage I + II / III + IV |
| Chondrosarcoma | 18 | 8/10 | 10/8 | 9/9 |
| MFH | 15 | 6/9 | 7/8 | 8/7 |
| Ewing's sarcoma | 15 | 0/15 | 6/9 | 6/9 |
| Leiomyosarcoma | 12 | 6/6 | 6/6 | 7/5 |
| Synovial sarcoma | 12 | 0/12 | 5/7 | 6/6 |
| Liposarcoma | 11 | 6/5 | 7/4 | 5/6 |
| Fibrosarcoma | 10 | 5/5 | 5/5 | 4/6 |
| Unclassified sarcoma | 10 | 6/4 | 7/3 | 5/5 |
| Rhabdomyosarcoma | 9 | 5/4 | 5/4 | 4/5 |
| Malignant schwannoma | 3 | 1/2 | 3/0 | 1/2 |

MFH = malignant fibrosis histiocytoma.

*© 2001 Cancer Research Campaign*

### Measurement of VEGF in tumour tissue sample

The concentration of VEGF in the tumour extract was measured by ELISA as described previously (Takano et al, 1996). Each well of a 96-well microtitre plate was sensitized with 100 μl of 10 mg $ml^{-1}$ of anti-human VEGF monoclonal antibody (mAb) (Dako) at room temperature for 1 hour. The plate was washed with PBS containing 0.05% Tween 20 and 0.1% bovine serum albumine (PBS-T-BSA). 100 μl per well of standard VEGF (0.1–1000 pg $ml^{-1}$: Dako) diluted in PBS-T-BSA or tissue sample diluted with an equal volume of PBS-T-BSA was added to the wells and incubated for 2 hours at 22˚C. After washing the wells 6 times, anti-human VEGF mAb (Sigma) used at a 1:1000 dilution in PBS-T-BSA was reacted in each well at 22˚C for 2 hours. The wells were washed 6 times, and then peroxidase-conjugated anti-IgG antibody (Dako) was added to each well and allowed to react for 2 hours at 37˚C. After a final wash, plates were incubated with 3.75 μmol $l^{-1}$ O-phenylenediamine, 1 mmol $l^{-1}$ $H_2O_2$ in 24 mmol $l^{-1}$ sodium citrate, 51 mmol $l^{-1}$ $Na_2HPO_4$, pH 5.0 for 15 minutes. The reaction was stopped by adding 100 μL 2 mol $l^{-1}$ $H_2SO_4$ and absorbance was read at 492 nm with a background subtraction at 620 nm. All standards and samples were measured in duplicate.

### Statistical analysis

Results were expressed as mean ± standard deviation (SD). The variances were analysed by the F test. Student's *t*-test (equal variance groups) and the Wilcoxon U test (unequal variance group) were used for statistical comparisons between 2 groups. Correlation coefficient was determined by Spearman's rank correlation test. A *P* value of less than 0.05 was considered to be statistically significant. Date of diagnosis was considered as the time of origin. For survival curves, we considered as an event, all deaths, whatever their cause. For the local recurrence-free interval, we considered strictly local recurrence as an event. For patients who died without any local recurrence, they were considered censored data at the time of death. For metastasis-free interval, we considered the first metastasis as an event. For patients who died without any metastasis, they were considered censored data at the time of death. The overall observed survival functions and probabilities were estimated using the Kaplan–Meier method. The log rank test was used to detect differences between survival curves for stratified variables. Univariate and multivariate analyses were performed using Cox's proportional hazard model. For multivariate analysis, VEGF concentration in the tumour tissue were separately adjusted to the clinocopathologic parameters through which the independent prognostic value of these parameters was determined.

### RESULTS

### MVD and VEGF concentration in tumour tissue

Photomicrograph of representative soft tissue sarcoma stained for factor VIII is shown in Figure 1. No significant association was observed between MVD and histologic type of soft tissue sarcomas (data not shown). In patients with soft tissue sarcoma, there was no statistical correlation between MVD and the VEGF concentration in the tumour tissue ($P$ = 0.075, r = 0.433).

### Levels of MVD and VEGF concentration in the tumour tissue in relation to clinical stage and histologic grade

A correlation between MVD and stage of the disease did not existed (Table 2). In contrast, the concentration of VEGF in the tumour tissue correlated with the clinical stage of the disease.

There was a significant correlation between the grade of histologic malignancy of the tumour and tissue VEGF concentration, but not with MVD. At higher grades, tissue VEGF concentration increased (Table 2).

### Correlation between MVD and VEGF concentration in the tumour tissue and clinical variables

The mean MVD in patients who developed local recurrence or metastasis in the patients who developed metastasis showed no significant difference compared with the mean MVD in those who

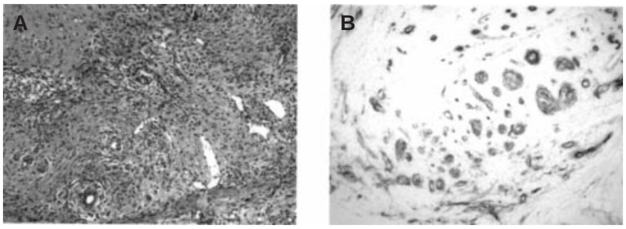

**Figure 1** Representative immunohistochemical staining for factor VIII in soft tissue sarcoma. (**A**) Haematoxylin and eosin staining (x 200). (**B**) Blood vessels were identified by staining endothelial cells for factor VIII (x 200)

**Table 2** Levels of MVD and VEGF concentration in relation to clinical stage and histologic grade in patients with soft tissue sarcoma

| Variable | Number of patients | MVD (/mm²) (mean ± SD) | VEGF concentration in the tumour tissue (μMol $mg^{-1}$ protein) (mean ± SD) |
|---|---|---|---|
| Stage | | | |
| I + II | 55 | 29.5 ± 10.4 | 1.7 ± 0.8 ]* |
| III | 32 | 33.1 ± 15.8 | 3.3 ± 1.4 ]** |
| IV | 28 | 35.4 ± 12.6 | 6.4 ± 2.4 ]* |
| Grade | | | |
| Low + Moderate | 43 | 32.5 ± 14.7 | 2.8 ± 1.4 ]* |
| High | 72 | 36.8 ± 14.8 | 4.9 ± 2.1 |

MVD = microvessel density; VEGF = vascular endothelial growth factor.
*$P$ < 0.05, **$P$ < 0.01, SD = standard deviation.

*© 2001 Cancer ResearchCampaign*

**Table 3** Levels of MVD and VEGF concentration in relation to clinical variables in patients with soft tissue sarcoma

| Variable | Number of patients | MVD (/mm²) (mean ± SD) | VEGF concentration in the tumour tissue (μMol mg⁻¹ protein) (mean ± SD) |
|---|---|---|---|
| Local recurrence (–) | 64 | 32.4 ± 11.8 | 1.9 ± 0.7 ]** |
| Local recurrence (+) | 51 | 35.6 ± 12.4 | 6.3 ± 1.9 |
| Metastasis (–) | 63 | 29.2 ± 16.3 | 2.3 ± 1.7 ]** |
| Metastasis (+) | 52 | 31.4 ± 14.5 | 4.9 ± 1.6 |

MVD = microvessel density; VEGF = vascular endothelial growth factor.
**$P < 0.01$; SD = standard deviation.

did not ($P$ = 0.087 and $P$ = 0.075, respectively) (Table 3). The mean tumour VEGF concentration in patients who developed either local recurrence or metastasis was higher than in those who did not develop the respective condition ($P < 0.001$ and $P = 0.004$, respectively) (Table 3).

### MVD, level of VEGF expression, and prognosis

In the present study, the MVD of patients with soft tissue sarcoma was 35.4 ± 11.2 /mm$^2$, and the tissue VEGF concentration was 2.48 ± 0.98 μMol mg$^{-1}$ protein. We divided the patients into those with high MVD (> 35/mm$^2$), and those with low MVD (≤ 35/mm$^2$), based on the median value. We also divided the patients into those with high tissue VEGF concentration (> 2.5 μMol mg$^{-1}$ protein), and those with low tissue VEGF concentration (≤ 2.5 μMol mg$^{-1}$ protein), also based on the median value. The prognosis of those in the high tissue VEGF concentration group was significantly worse than those in the low tissue VEGF concentration group (Figure 2). However, there was no significant difference in the prognosis between the high MVD group and the low MVD group.

### Univariate and multivariate analyses

Table 4 shows the results of the univariate analysis for prognostic parameters for overall survival, the local recurrence-free and metastasis-free survival. In univariate analysis, tumour size, clinical stage, grade of histologic malignancy, tissue VEGF concentration had a significant impact on overall mortality and metastasis ($P < 0.01$). Clinical stage, grade of histologic malignancy, and tissue VEGF concentration significantly affected local recurrence-free survival ($P < 0.01$), and there was a trend ($P = 0.05$) for 2 variables: tumour size and localization (Table 4). Univariate analysis resulted in identification of VEGF concentration in the tumour tissue, which had an association with the overall survival, the local recurrence and metastasis of the patients.

The effects of variables presumably associated with prognosis were studied by multivariate analysis. Tissue VEGF concentration was significantly correlated with a poorer overall survival, the local recurrence-free and metastasis-free survival (Table 5). The parameter emerged as an independent prognostic parameter.

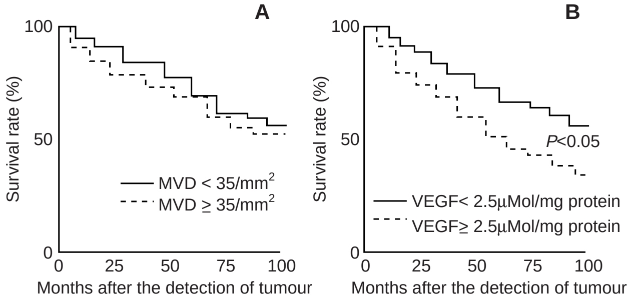

**Figure 2** (**A**) Overall survival curves according to high and low microvessel density (MVD) in the tumour tissue of patients with soft tissue sarcoma. (**B**) Overall survival curves according to high and low concentration of vascular endothelial growth factor (VEGF) in the tumour tissue of patients with soft tissue sarcoma

### DISCUSSION

We hypothesized that neovascularity as well as angiogenic factor involved in its regulation play a role in determining the prognosis of soft tissue sarcomas. We found that the concentration of VEGF, but not microvessel count, in the tumour tissue was significantly correlated with the prognosis, stage of disease, and histologic grade of soft tissue sarcomas. In addition, tumours associated with local recurrence or metastasis showed a significantly higher tissue VEGF concentration than those without either.

Recent independent studies of a large series of soft tissue sarcomas showed that histologic tumour grade, tumour depth and tumour size were independent prognostic factors (Gaynor et al, 1992; Coindre et al, 1996). In the current study, Cox proportional hazard multivariate analysis revealed that histologic grade was the most valuable independent prognostic factor in patients with soft tissue sarcoma. Moreover, tissue VEGF concentration measured by ELISA was also an independent prognostic factor for patients with soft tissue sarcomas. Similar results have been reported in studies on several cancers including human colon cancer (Takahashi et al, 1995), brain tumour (Takano et al, 1996), and gastric cancer (Maeda et al, 1996).

However, in the present study, the count of microvessels stained with factor VIII antibody was not correlated with the prognosis of soft tissue sarcomas. Generally speaking, neovascularity assessed by the microvessel counting in the tumour tissue has a prognostic value in a variety of cancers (Yamazaki et al, 1994; Maeda et al, 1995; Brawer, 1996; Gasparini et al, 1996; Takabayashi et al, 1996). In addition, the density of microvessels was not correlated with the tissue VEGF concentration measured by ELISA in soft tissue sarcomas. Microvessel counting in the tumour tissue may not accurately represent the angiogenic capacity in soft tissue sarcomas. Other techniques may be necessary to predict the neoangiogenesis of soft tissue sarcomas.

© 2001 Cancer Research Campaign

**Table 4** Univariate analysis of clinicopathologic parameters and MVD and tissue VEGF concentration in relations to overall survival, local recurrence-and metastasis-free survival in patients with soft tissue sarcoma

| Parameters | Number of patients | Overall 5-year survival rate (%) | *P* value | Recurrence-free 5-year survival rate (%) | *P* value | Metastasis-free 5-year survival rate (%) | *P* value |
|---|---|---|---|---|---|---|---|
| Age (year) | | | | | | | |
| < 50 | 55 | 69.8 | 0.4 | 62.5 | 0.6 | 68.4 | 0.5 |
| ≥ 50 | 60 | 63.5 | | 65.5 | | 65.6 | |
| Sex | | | | | | | |
| Male | 58 | 65.2 | 0.9 | 64.6 | 0.7 | 63.5 | 0.9 |
| Female | 57 | 64.8 | | 61.6 | | 65.9 | |
| Tumour size | | | | | | | |
| < 5 cm | 61 | 83.4 | < 0.001 | 75.4 | 0.05 | 77.4 | < 0.001 |
| ≥ 5 cm | 54 | 54.5 | | 65.3 | | 53.5 | |
| Localization | | | | | | | |
| extremity | 68 | 61.5 | 0.08 | 69.4 | 0.05 | 65.4 | 0.6 |
| trunk | 47 | 58.1 | | 58.9 | | 64.6 | |
| Stage | | | | | | | |
| I + II | 55 | 85.3 | < 0.001 | 78.3 | 0.003 | 75.2 | 0.002 |
| III + IV | 60 | 50.6 | | 49.2 | | 42.8 | |
| Grade | | | | | | | |
| Low + Moderate | 43 | 76.5 | 0.001 | 86.2 | < 0.001 | 71.3 | < 0.001 |
| High | 72 | 48.5 | | 58.6 | | 40.2 | |
| MVD ($mm^2$) | | | | | | | |
| < 35 | 58 | 64.5 | 0.4 | 67.3 | 0.2 | 57.3 | 0.4 |
| ≥ 35 | 57 | 60.2 | | 62.5 | | 50.9 | |
| VEGF ($\mu$Mol $mg^{-1}$ protein) | | | | | | | |
| < 2.5 | 63 | 66.6 | < 0.001 | 71.5 | < 0.001 | 66.5 | 0.003 |
| ≥ 2.5 | 52 | 40.2 | | 47.5 | | 46.5 | |

MVD = microvessel density; VEGF = vascular endothelial growth factor.

**Table 5** Multivariate analysis for overall survival, local recurrence and metastasis

| | Cox's hazard ratio (95% CI) and *P* value | | |
|---|---|---|---|
| **Parameters** | **Overall survival** | **Local recurrence** | **Metastasis** |
| Tissue VEGF concentration | 1.94 (1.03–3.60) 0.025 | 1.32 (1.34–3.90) 0.001 | 1.13 (1.09–1.18) < 0.001 |
| Tumour size | 1.08 (1.03–1.12) 0.255 | 1.01 (0.99–1.13) 0.395 | 1.02 (0.98–1.08) 0.355 |
| Clinical stage | 6.92 (2.37–20.12) 0.007 | 2.56 (0.95–6.95) 0.067 | 1.72 (0.72–4.04) 0.224 |
| Grade of histologic malignancy | 2.44 (0.95–6.85) 0.019 | 2.10 (0.43–8.85) 0.378 | 1.87 (0.61–5.74) 0.254 |

VEGF = vascular endothelial growth factor; CI = confidence interval.

In our other study, the level of bFGF expression was not correlated with the microvessel density in soft tissue sarcomas, although bFGF expression was observed in the tumour tissue (data not shown). Our data was consistent with the findings of Takahashi et al, who demonstrated that no significant correlation was observed between the expression of bFGF and the microvessel density in colon cancer. Similar results have also been reported in gastric cancer (Takahashi et al, 1996). The reason why the level of VEGF or bFGF expression did not correlate with the density of microvessels in soft tissue sarcomas still remains unclear. Further studies are needed to clarify this discrepancy in soft tissue sarcomas. Angiogenic factors secreted from tumours may contribute to all processes of tumour advancement not only via a paracrine pathway to surrounding microvessels, but also via an autocrine pathway to tumour cells (Berkman et al, 1993; Boocock et al, 1995). To confirm the hypothesis that VEGF or bFGF plays a role in the tumour aggressiveness and the neoangiogenesis in soft tissue sarcomas, it must be demonstrated that receptors for each factor are present on tumour cells and tumour endothelia. We are currently studying the significance of the level of expression of VEGF and bFGF and their receptors in soft tissue sarcomas.

In the present study, in the sera from soft tissue sarcoma patients, VEGF levels were within normal range, even in the case with extremely high levels of VEGF in the tumour tissue (data not shown). Failure to detect VEGF in the sera from soft tissue sarcoma, as well as several types of cancer, might be explained by rapid binding of VEGF to cell receptors or to extracellular matrix, resulting in the clearance of VEGF from the circulation (Yeo et al, 1993).

It is difficult to choose a histological site to study microvessel count and VEGF expression in soft tissue sarcomas, because a solid tumour such as a soft tissue sarcoma does not occur in a single layer of the bowel except in its earliest stages. Several studies have demonstrated that microvessel density at the invasive

*© 2001 Cancer Research Campaign*

edge is significantly higher than that within the tumour, and that the highest level of angiogenic activity occurs at the invasive edge of the tumour (Takahashi et al, 1995; Takano et al, 1996). We also found in another in vivo study that hot spots of microvessels were observed at the invasive edge of the tumour tissue in tumour-bearing mice; a significant correlation existed between the level of expression of VEGF at the invasive edge, and tumour growth and metastasis (data not shown). These findings suggest that the invasive edge of the tumour is the most active area in local invasion, as well as metastasis. In the current study, we chose the highest density of microvessels in the tumour tissue.

The current study provides evidence to support the hypothesis that the concentration of VEGF in the tumour tissue is strongly linked with disease outcome and with the prognosis of patients with soft tissue sarcomas. If VEGF expression in the tumour tissue proves to be reliable prognostic factors, patients at high risk for developing local recurrence or metastasis, can be selected for adjuvant therapy. It has been reported that anti-VEGF neutralizing antibody inhibits angiogenesis and tumour growth in vitro and in an in vivo tumour model (Borgstrom et al, 1996; Presta et al, 1997). If VEGF is responsible for tumour angiogenesis in soft tissue sarcomas, therapeutic strategies using either specific antibodies or antisense RNA to VEGF, may inhibit tumour angiogenesis.

In conclusion, the correlation of tissue VEGF concentration and disease outcome suggests that angiogenesis may be a potential prognostic marker in soft tissue sarcomas. In addition, VEGF may provide a potential target for therapy in soft tissue sarcomas.

### REFERENCES

Berkman RA, Merrill MJ, Reinhold WC, Monacci WT, Saxena A and Clark WC et al (1993) Expression of vascular permeability factor/vascular endothelial growth factor gene in central nervous system neoplasms. *J Clin Inves* **91**: 153–159

Boocock CA, Charnock-Jones DS, Sharkey AM, McLaren J, Barker PJ and Wright KA et al (1995) Expression of vascular endothelial growth factor and its receptor fit and KDR in ovarian carcinoma. *J Natl Cancer Inst* **87**: 506–516

Borgstrom P, Hillan KJ, Sriramarao P and Ferrara N (1996) Complete inhibition of angiogenesis and growth of microtumors by anti-vascular endothelial growth factor neutralizing antibody: novel concepts of angiogenesis therapy from intravital videomicroscopy. *Cancer Res* **56**: 4031–4039

Brawer MK (1996) Quantitative microvessel density: a staging and prognostic marker for human prostatic carcinoma. *Cancer* **78**: 345–349

Claffey KP, Brown LF, del Aguila LF, Tognazzi K, Yeo KT and Manseau EJ et al (1996) Expression of vascular permeability factor/vascular endothelial growth factor by melanoma cells increases tumor growth, angiogenesis, and experimental metastasis. *Cancer Res* **56**: 172–181

Coindre JM, Terrier P, Bui NB, Bonichon F, Colli F and Le Doussal V et al(1996) Prognostic factors in adults patients with locally controlled soft tissue sarcoma: A study of 546 patients from the French federation of cancer centers sarcoma group. *J Clin Oncol* **14**: 869–877

Folkman J (1986) How is blood vessel growth regulated in normal and neoplastic tissue? *Cancer Res* **46**: 467–473

Folkman J, Watson K, Ingber D and Hanahan D (1989) Induction of angiogenesis during the transition from hyperplasia to neoplasia. *Nature* **338**: 58–61

Folkman J (1990) What is the evidence that tumors are angiogenesis dependent? *J Natl Cancer Inst* **82**: 4–6

Gasparini G, Bonoldi E, Viale G, Verderio P, Boracchi P and Panizzoni GA et al(1996) Prognostic and predictive value of tumor angiogenesis in ovarian carcinomas. *Int J Cancer* **69**: 205–211

Gaynor JJ, Tan CC, Casper ES, Collin CF, Friedrich C and Shiu M et al (1992) Refinement of clinicopathologic staging for localized aoft tissue sarcoma of the extremity: A study of 423 adults. *J Clin Oncol* **10**: 1317–1329

Gimbrone MA, Leapman S, Cotran RS and Folkman J (1972) Tumor dormancy in vivo by prevention of neovascularization. *J Exp Med* **136**: 261–276

Gimbrone MA, Cotran RS, Leapman S and Folkman J (1974) Tumor growth neovascularization: an experimental model using rabbit cornea. *J Natl Cancer Inst* **52**: 413–427

Hermanek P and Sobin LH, editors (1992) *UICC TNM Classification of malignant tumours*. 4th ed. 2nd rev. Berlin: Springer-Verlag

Liotta L, Kleinman J and Saldel G (1974) Quantitative relationships of intravascular tumor cells, tumor vessels, and pulmonary metastases following tumor implantation. *Cancer Res* **34**: 997–1004

Liotta LA, Steeg PS and Stetler-Stevenson WG (1991) Cancer metastasis and angiogenesis: an imbalance of positive and negative regulation. *Cell* **64**: 327–336

Maeda K, Chung Y-S, Takatsuka S, Ogawa Y, Sawada Y and Yamashita Y et al (1995) Tumor angiogenesis as a predictor of recurrence in gastric carcinoma. *J Clin Oncol* **13**: 477–481

Maeda K, Chung Y-S, Ogawa Y, Takatsuka S, Kang S-M and Ogawa Masafumi et al (1996) Prognostic value of vascular endothelial growth factor expression in gastric carcinoma. *Cancer* **77**: 858–863

Mandard AM, Petiot JF, Marnay J, Mandard JC, Chasle J and de Ranieri E et al (1989) Prognostic factors in soft tissue sarcomas. A multivariate analysis of 109 cases. *Cancer* **63**: 1437–1451

Miller JW, Adamis AP, Shima DT, D'Amore PA, Moulton, RS and O'Reilly MS et al (1994) Vascular endothelial growth factor/vascular permeability factor is temporally and spatially correlated with ocular angiogenesis in a primate model. *Am J Pathol* **145**: 574–584

Presta LG, Chen H, O'Connor SJ, Chisholm V, Meng G and Krummen L et al (1997) Humanization of an anti-vascular endothelial growth factor monoclonal antibody for therapy for solid tumors and other disorders. *Cancer Res* **57**: 4593–4599

Takabayashi Y, Akiyama S, Yamada K, Akiba S and Aikou T (1996) Angiogenesis as an unfavorable prognostic factor in human colorectal carcinoma. *Cancer* **78**: 226–231

Takahashi Y, Kitadai Y, Bucana CD, Cleary KR and Ellis LM (1995) Expression of vascular endothelial growth factor and its receptor, KDR, correlates with vascularity, metastasis, and proliferation of human colon cancer. *Cancer Res* **55**: 3964–3968

Takahashi Y, Cleary KR, Mai M, Kitadai Y, Bucana CD and Ellis LM (1996) Significance of vessel count and vascular endothelial growth factor and its receptor (KDR) in intestinal-type gastric cancer. *Clin Cancer Res* **2**: 1679–1684

Takano S, Yoshii Y, Kondo S, Suzuki H, Maruno T and Shirai S et al (1996) Concentration of vascular endothelial growth factor in the serum and tumor tissue of brain tumor patients. *Cancer Res* **56**: 2185–2190

Yamazaki K, Abe S, Takekawa H, Sudou N, Watanabe N and Ogura S et al (1994) Tumor angiogenesis in human lung adenocarcinoma. *Cancer* **74**: 2245–2250

Yeo K, Wang HH, Nagy JA, Sioussat TM, Ledbetter SR and Hoogewerf AJ et al (1993) Vascular permeability factor (vascular endothelial growth factor) in guinea pig and human tumor and inflammatory effusions. *Cancer Res* **53**: 2912–2918

Zhang HT, Craft P, Scott PA, Ziche M, Weich HA, Harris AL and Bicknell R (1995) Enhancement of tumor growth and vascular density by transfection of vascular endothelial cell growth factor into MCF-7 human breast carcinoma cell. *J Natl Cancer Inst* **87**: 213–217

© 2001 Cancer Research Campaign
